# NAT10 as a potential prognostic biomarker and therapeutic target for HNSCC

**DOI:** 10.1186/s12935-021-02124-2

**Published:** 2021-08-06

**Authors:** Wenjie Tao, Guocai Tian, Shengming Xu, Jiayi Li, Zhiyuan Zhang, Jiang Li

**Affiliations:** 1 Department of Oral and Maxillofacial-Head Neck Oncology, Shanghai Ninth People’s Hospital, College of Stomatology, Shanghai Jiao Tong University School of Medicine, Shanghai, People’s Republic of China; 2 National Clinical Research Center for Oral Diseases, Shanghai, People’s Republic of China; 3 Shanghai Key Laboratory of Stomatology & Shanghai Research Institute of Stomatology, Shanghai, People’s Republic of China; 4 Research Unit of Oral and Maxillofacial Regenerative Medicine, Chinese Academy of Medical Sciences, Shanghai, People’s Republic of China; 5 Department of Oral Pathology, Ninth People’s Hospital, Shanghai Jiao Tong University School of Medicine, Shanghai, People’s Republic of China

**Keywords:** HNSCC, mRNA modification regulator, Prognosis, NAT10, Biomarker, Therapeutic target

## Abstract

**Background:**

Increasing evidence has demonstrated the critical roles of mRNA modification regulators on multiple types of cancers. However, it is still poorly known about the prognostic and therapeutic value of mRNA modification regulators in HNSCC.

**Methods:**

The gene expression profile of 36 mRNA modification regulators and their corresponding clinical data were obtained from The Cancer Genome Atlas (TCGA) and Gene Expression Omnibus (GEO). Stepwise regression in R with both directions was used to construct a model for the prognosis of HNSCC. Univariate Cox regression survival analysis was performed to identify the most significant risk gene. Gene set enrichment analysis (GSEA) was applied to determine the cancer-associated pathways with NAT10. Immunohistochemistry (IHC) staining was performed to evaluate the expression of NAT10 in formalin fixed paraffin-embedded (FFPE) samples of HNSCC. Univariate and multivariate Cox regression survival analysis performed to identify the independent risk factors associated with the OS of patients with HNSCC. HNSCC cell lines (Cal-27, FaDu, and Detroit-562) were transfected with short interfering RNA (siRNA) targeting NAT10 or treated with Remodelin, a small-molecule inhibitor of NAT10. Knockdown efficiency of siRNA was assessed by quantitative real-time PCR (qRT-PCR) and western blotting. In addition, CCK-8 assay, scratch assay and transwell assay were used to examine the proliferation, migration, and invasion abilities of the three HNSCC cell lines after NAT10 was inhibited genetically and pharmaceutically. Cell cycle and cell apoptosis assays were performed by flow cytometry. Finally, the therapeutic value of Remodelin in HNSCC was evaluated via a patient-derived xenograft (PDX) model. The statistical analysis was performed with SPSS 23.0.

**Results:**

A risk prediction model containing 10 mRNA modification regulators was constructed and showed prognostic value in HNSCC. NAT10 was further identified as a key risk gene and independent prognostic factor in TCGA HNSCC dataset. The GSEA analysis suggested that high NAT10 expression was associated with MYC, E2F, G2M checkpoint, mTORC1, DNA repair and oxidative phosphorylation pathways. NAT10 protein expression was significantly up-regulated in tumour cells compared to normal epithelial cells in FFPE samples and increased NAT10 protein expression was correlated with poor overall survival of 267 HNSCC patients. Genetic depletion of NAT10 using siRNA or chemical inhibition of NAT10 using Remodelin resulted in reduced cell proliferation, migration and invasion abilities in Cal-27, FaDu and Detroit-562 cells. Knockdown of NAT10 using siRNA significantly increased cell cycle arrest in S/G2-phase. Remodelin significantly inhibited tumour growth and tumour cell proliferation in the PDX model of HNSCC.

**Conclusions:**

NAT10 could be a potential prognostic marker and a therapeutic target for HNSCC.

**Supplementary Information:**

The online version contains supplementary material available at 10.1186/s12935-021-02124-2.

## Background

Head and neck squamous cell carcinoma (HNSCC), arising from the lip, oral cavity, oropharynx, hypopharynx and larynx, is the eighth most frequent cancer worldwide, accounting for almost 800,000 new cases and approximately 390,000 cancer-related deaths in 2020 [1]. Despite significant advances in HNSCC treatments including surgery, chemotherapy, radiotherapy, targeted therapy and immunotherapy, the 5-year survival rate and long-term prognosis of these patients have not dramatically improved in recent decades [2, 3]. HNSCC is a remarkably heterogeneous malignant tumour characterized by biological and clinical heterogeneity [4]. Currently, the generally used clinical classification system for HNSCC patients is TNM staging, however the patients with the same anatomical site and TNM stage differ in prognosis and clinical outcomes [5]. Tobacco and alcohol consumption are the classical risk factors for HNSCC [6]. Recently, human papilloma virus (HPV) infection has become the most recognized key factor in HNSCC [7]. In addition, numerous molecular and clinical risk factors have been investigated with limited clinical utility [8]. Thus, new prognostic biomarkers, therapeutic targets and treatment strategies for patients with HNSCC are urgently needed.

It is well accepted that gene expression is regulated in a complex manner through genetic and epigenetic control [9]. Genetic abnormalities, including gene mutation, deletion, amplification, or chromosomal translocation, and abnormal epigenetic alterations, such as DNA or histone modification, may lead to the development of human diseases, including cancers [10, 11]. Recent advances in mRNA modification have opened an entirely new layer of gene expression regulation in human cancer [12, 13]. Growing evidence indicates that mRNA modifications and associated regulators contribute to the initiation, progression, and drug response of cancers [12, 14]. Only a few types of mRNA modifications have been revealed, such as N6-methyladenosine (m6A), N1-methyladenosine (m1A), 5-methylcytosine (m5C), 3-methylcytosine (m3C), pseudouridine (Ψ) and N4-acetylcytidine (ac4C) [15–18]. The turnover of each mRNA modification is regulated by related enzymes called regulators, such as METTL3–METTL14 as the methyltransferase for m6A, FTO as the demethylase for m6A and YTHDC2 as the m6A reader [19]. Currently, the roles of mRNA modification regulators in cancer is still poorly understood, especially in HNSCC. Moreover, the prognostic and therapeutic potential of mRNA modification regulators for HNSCC remain unclear.

In this study, we analyzed 36 mRNA modification regulators and constructed a risk prediction model containing 10 potential prognostic risk signatures in The Cancer Genome Atlas (TCGA) HNSCC dataset. Of the candidate genes in these potential prognostic risk signatures, NAT10 was the most promising prognostic risk gene in the TCGA and GEO datasets. The HNSCC dataset from TCGA revealed that the highly expressed NAT10 gene is an independent prognostic factor of HNSCC. IHC analyses revealed high protein expression of NAT10 in HNSCC FFPE samples, which indicated a poor overall survival rate in 267 HNSCC patients. In addition, NAT10 plays an essential role in regulating the proliferation, migration, invasion and cell cycle of tumour cells, thus promoting tumour growth and progression. Remodelin, a small-molecule inhibitor of NAT10, significantly suppressed the growth of HNSCC in a patient-derived xenograft (PDX) model, indicating that targeting NAT10 may be a new therapeutic strategy for HNSCC patients.

## Methods

### Dataset acquisition and screening of mRNA modification regulators

TCGA (https://www.cancer.gov/tcga.) is a public database containing clinical information and RNA-seq results of HNSCC patients, which is widely used for cancer research [20]. Our data were downloaded from TCGA through the FireBrowse (http://firebrowse.org/?cohort=HNSC&download_dialog=true#) website. Data were obtained for 566 samples (520 were primary HNSCC samples, two were metastatic samples and 44 were normal samples) with clinical and RNA-seq information. In addition, Gene Expression Omnibus (GEO) contains many datasets that could be used for data mining [21]. The GSE65858 dataset contains 270 samples with complete clinical and mRNA expression data was downloaded. RNA-seq data of 36 mRNA modification regulators were selected and visualized in a heatmap by R software (version 3.6.3). Tumour stage, age, clinical T, N and M stage, gender, pathological grade and modification type were considered for heatmap clustering.

### Construction of a risk prediction model for HNSCC

Stepwise regression in R with both directions in the mRNA modification regulator gene list based on the TCGA HNSCC cohort was used to construct a model for the prognosis of HNSCC, and 10 risk genes associated with the prognosis of HNSCC were screened. Pearson correlation analysis was performed among the 10 genes. The risk score of each gene was calculated by multiplying the expression of the gene and its coefficient as reported previously [22]. In addition, the risk score for each patient was defined as the sum of each gene’s score. Patients were divided into a high-risk group and a low-risk group based on the median value of their risk score. The expression levels of the 10 risk genes in the high-risk group and low-risk group of HNSCC as well as normal samples were visualized. The distributions of the risk scores and overall survival status were visualized, and the Kaplan–Meier method was applied to generate overall survival (OS) curves for the high- and low-risk groups. The log-rank test was used to evaluate statistical significance. To identify genes correlated with OS, univariate Cox regression survival analysis was performed, the hazard ratio and P values were calculated, and a forest plot was used to display the results.

### Gene set enrichment analysis (GSEA) of NAT10

GSEA is used to explore the consistency and variability of phenotypes to find common or different biofunctions [23]. GSEA software (version 4.0.3, https://www.gsea-msigdb.org/gsea/downloads.jsp) was applied to identify the pathways associated with NAT10 in HNSCC. The 520 samples were divided into high and low expression groups based on the expression of NAT10, the median expression level of NAT10 was set as the cut-off, and there were 260 samples in each group. The files needed to run GSEA were made according to the help information of the GSEA website, and the predefined “hallmark gene sets” were used for analyses. The normalized enrichment score (NES) was obtained by running GSEA, and the cut-off for significance was defined as follows: a nominal P-value of < 0.05 and FDR q-value of < 0.05.

### H&E and IHC staining to detect the expression of NAT10 in 267 HNSCC samples

HNSCC samples of 267 patients (215 patients diagnosed with oral squamous carcinoma and 52 diagnosed with oropharyngeal squamous carcinoma) were obtained from Shanghai 9th People’s Hospital with approval from the ethics committee and informed consent was obtained from the patients. Haematoxylin and eosin (H&E) and the immunohistochemistry (IHC) staining of NAT10 (NAT10 antibody, Ab194297, Abcam, 1:1000 dilution) of samples were performed. For HE and IHC staining, the procedure followed a protocol reported previously [24]. The expression of NAT10 in tumours was estimated by two professional pathologists and defined as follows: high expression: cytoplasm or nucleus was stained dark brown; low expression: cytoplasm or nucleus was stained light brown, and no expression: cytoplasm or nucleus was not stained.

### Survival analysis based on 267 HNSCC patients

The gender, age, tumour site, grade, tumour stage, HPV status, tobacco and alcohol use, and primary or recurrent tumour status of 267 patients were collected. Phone calls were used to follow up the survival information of patients. Univariate and multivariate Cox regression survival analysis was performed to identify the independent risk factors associated with the OS of patients. Hazard ratios and p values were calculated by SPSS 23.0. For overall survival analyses, the log-rank test was performed, and a p value of < 0.05 was considered statistically significant.

### Construction of short interfering RNA (siRNA) and transfection of HNSCC cell lines

The siRNAs targeting NAT10 were constructed (Hanbio Biotechnology Co., Ltd., Shanghai). The sequences were as follows: si-NAT10-1 (5ʹ-GCACCACUGCUGAGAAUAATT-3ʹ), si-NAT10-2 (5ʹ-GCUCCUCAAGUUCUGGAAATT-3ʹ), si-NAT10-3 (5ʹ-GCAUGGACCUCUCUGAAUATT-3ʹ) and si-Scramble (5ʹ-UAUUCAGAGAGGUCCAUGCTT-3ʹ). 5-Carboxyfluorescein (FAM) was applied to trace siRNA in transfected Cal-27, FaDu and Detroit-562 cells. Cal-27 cells were purchased from the American Type Culture Collection (ATCC). FaDu and Detroit-562 cells were purchased from the National Collection of Authenticated Cell Cultures. Cell lines were confirmed to be free of contamination by short tandem repeat analysis. To verify the efficiency of NAT10 downregulation by siRNA, tumour cells were seeded in 6-well plates. 40 nM siRNAs and 20 µL siRNA Fit solution were added to each well. After culture for 6–17 h, the medium was removed and replaced with fresh complete medium. The cells were collected at 48 h after transfection for reverse transcription polymerase chain reaction and western blotting.

### Quantitative real-time PCR (qRT-PCR)

Total RNA of the siRNA- and si-scramble-transfected cells was obtained, ReverTra Ace^®^ qPCR RT Kit (TOYOBO) was used for reverse transcription. SYBR Green Real-time RCR Master Mix (TOYOBO) was applied for quantitative real-time PCR analysis according to the instructions. The primers were designed as follows: NAT10(F: GCAGCCACAAACATTCGCTA; R: AGGAGGATGACCACTAGCCC). The primers for the standard internal gene were designed as follows: GAPDH(F: TCAAGGCTGAGAACGGGAAG; R: TCGCCCCACTTGATTTTGGA); The primers of the top 10 ranked genes in MYC targets were as follows: LDHA (F: TAGCAGATTTGGCAGAGAGTAT; R: CAAGGAACACTAAGGAAGACAT); MYC (F: CGGAAACGACGAGAACAGT; R: CATAGGTGATTGCTCAGGACAT); HSPD1 (F: CACTCGTCTTGAATAGGCTAAA; R: AATCCCTCTTCTCCAAACACT); CDC20 (F: ATTCCTTCCCTGCCAGAC; R: GCCAGTACATTCCCAGAACTC); RRP9 (F: CGTGAAGGTGTGGAATGT; R: GTCCTGGTGTCCGAAGA); ODC1 (F: ATGTGAATGATGGCGTCTAT; R: ACTTCTCATCTGGTTTAGGTCTC); NOP16 (F: CTCGGGACCTCATTGACTAT; R: GTCACTCCACCTCCATCTTC); NOLC1 (F: AAAGGCGGCAGTGGTAGT; R: GGAAATGTGTTAGGGGTCTGA); KPNA2 (F: GCCCGTCTTCACAGATTC; R: GCTCCACATTGACCTCTATTC); PHB (F: GTAGGGGAAGGGACTCATT; R: GCAGTGTGATGTTGACATTCT); b-actin (F: AAGGTGACAGCAGTCGGTT; R: TGTGTGGACTTGGGAGAGG). The primers were provided by BioSune Biotechnology Co., Ltd., Shanghai. The reaction procedures were as follows: 95 °C, 180 s; 94 °C, 15 s; 60 °C, 30 s; 72 °C, 30 s. The melting curve procedures were 95 °C, 10 s; 65 °C, 60 s; 97 °C, 1 s.

### Western blotting

Western blotting was applied to verify the expression of NAT10 in tumour cells at the protein level after transfection with siRNA. Total protein was extracted and the concentration was measured. Twenty micrograms of the protein of each sample were loaded for sodium dodecyl sulfate polyacrylamide gel electrophoresis (SDS-PAGE) and subsequently transferred to polyvinylidene fluoride (PVDF) membranes. Then, the membranes were blocked with 5% skim milk and TBST was used for membranes washing. Primary antibody (NAT10 antibody, Ab194297, Abcam, 1:1000 dilution; GAPDH antibody: 5174s, CST) was incubated in 4 °C overnight, followed by incubation with the horseradish peroxidase-conjugated (HRP) secondary antibodies for 1 h at room temperature. Finally, the detection of the immunoreactive proteins were visualized by enhanced chemiluminescence (ECL, Millipore) according to the manufacturer's instructions.

### In vitro studies

Three cell lines mentioned above were used for in vitro studies. For the cell proliferation assay, cells were pretreated with NAT10 siRNA or NAT10 inhibitor Remodelin hydrobromide [25] (Product ID: S7641, Selleck) at a concentration range of 5–60 μM. A total of 3000 cells were seeded in 96-well plates, and Cell Counting Kit-8 (CCK-8, Dojindo, Japan) was diluted in DMEM (1:10) to measure the optical density (OD) value at 450 nm on a multimode microplate reader (SpectraMax i3, Molecular Devices).

To determine the migration and invasion ability of the tumour cells, the three cell lines were transfected with siRNA or treated with Remodelin for 48 h. Cells were seeded into 6-well plates, and a scratch assay was performed with 10-μL pipette tips. Serum-free Dulbecco’s modified Eagle’s medium (DMEM) was added. After culturing for 24 h, pictures were taken, and ImageJ was used to calculate the area without cells. In addition, 10^5^ cells were seeded in the apical chamber of the transwell in 24-well cell culture plates with serum-free DMEM, and DMEM containing 20% FBS was added to the basolateral chamber of the transwell. After culturing for 48 h, 4% paraformaldehyde (PFA) was used for fixation, and crystal violet staining was performed. For the invasion assay, the apical chamber of the transwell was precoated with Matrigel. The other procedures were the same as mentioned above.

Flow cytometry was used to explore the changes in the cell cycle and cell apoptosis after transfection with NAT10 siRNA for 48 h, and scrambled siRNA was used as a control. For the cell cycle assay, cells were fixed with 70% alcohol overnight and then stained with mixed stain buffer (50 μg/mL propidium iodide (PI), 100 μg/mL RNase A, 0.2% Triton X-100) at 4 °C for 30 min. For the apoptosis assay, cells were collected and stained according to the manufacturer’s instructions for the FITC Annexin V apoptosis detection kit (Product ID: 556547, BD Pharmingen). BD FACSCalibur was used to detect positive events, and FlowJo (version 10.6.2) was used for data analysis.

### Evaluation of the therapeutic value of Remodelin via the PDX model

BALB/c nude mice (6 weeks, male, approximately 20 g) were obtained from Shanghai Sippr-BK Laboratory Animal Co. Ltd. and maintained in specific-pathogen-free facilities at Shanghai Ninth People’s Hospital. All experimental procedures were approved by the Laboratory Animal Care and Use Committees of the hospital. PDX model was constructed as we reported previously [24]. Each mouse was anesthetized with inhalation of 2–4% isoflurane/O_2_ for less than 30 min, then tumour tissues from patients were cut into 20–30 mm^3^ pieces and seeded in the flanks of mice. When the tumour volume reached 1500–2000 mm^3^, the mice were sacrificed. Tumour tissues were cut into pieces and seeded again for passaging. The expression of NAT10 (NAT10 antibody, Ab194297, Abcam, 1:1000 dilution) and Ki-67 (Ki-67 antibody, Ab927742, Abcam, 1:1000 dilution) in the PDX model were evaluated by IHC. Third-passage mice with tumour volumes of 150–250 mm^3^ were used to evaluate the therapeutic value of the NAT10 inhibitor (Remodelin). Remodelin was dissolved in dimethyl sulfoxide (DMSO) and diluted in sterile Tween 80 and 45% 2-hydroxypropyl-b-cyclodextrin solution [26]. The final formulation was 20% DMSO, 65% (45% 2-hydroxypropyl-b-cyclodextrin solution) and 15% Tween 80. The inhibitor and corresponding vehicle were administered intragastrically daily at 100 mg/kg/day. Tumour volume (TV) was recorded every 3–5 days, and mice were sacrificed after therapy for 4 weeks. The tumour growth inhibition rate (TGI) was calculated as

$$ {\text{TGI}} = \left( {{\text{TV}}_{{{\text{vehicle}}}} - {\text{TV}}_{{{\text{treatment}}}} } \right)/\left( {{\text{TV}}_{{{\text{vehicle}}}} - {\text{TV}}_{{{\text{initial}}}} } \right) \times 100\% . $$

### Statistical analyses

SPSS 23.0 was used for statistical analyses, and GraphPad Prism 9.0.2 was applied to generate figures. For survival analyses, the log-rank test was performed, and a P-value of < 0.05 was considered to indicate a significant difference.

In vitro and in vivo results are presented as means ± SD (standard deviation). A t test was used to compare two groups, and one-way ANOVA was used to compare three or more groups. Two-way ANOVA was used to evaluate the therapeutic value of Remodelin, and P-values < 0.05 were considered to indicate statistical significance.

## Results

### The constructed risk prediction model showed prognostic value in HNSCC

**Fig. 1:**
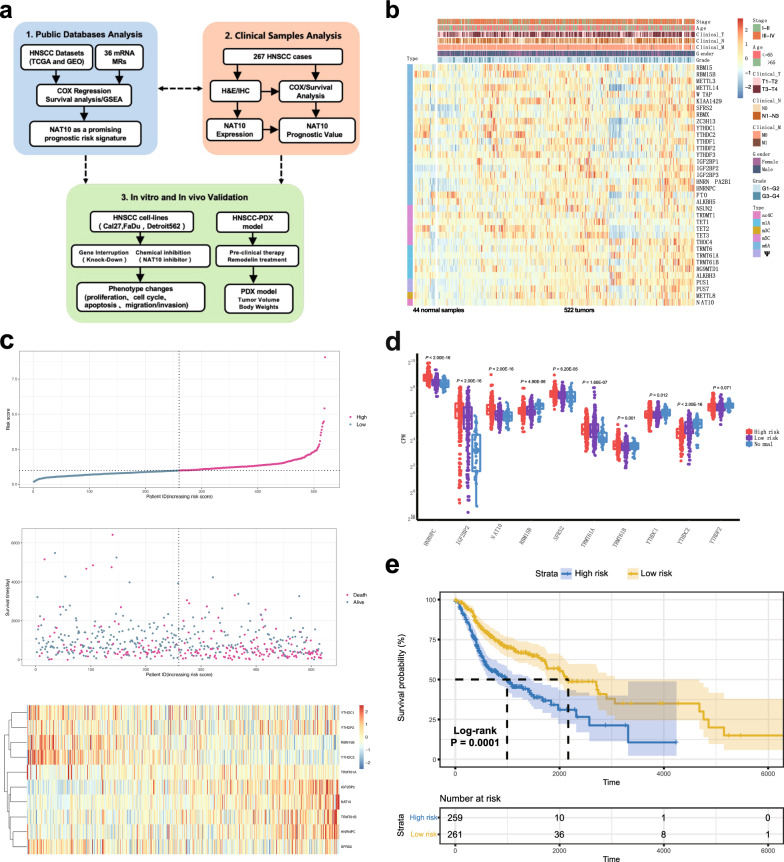
A risk prediction model showed prognostic value for HNSCC, data from the TCGA. **a** A workflow chart of the study (*MRs* modification regulators). **b** The RNA expression of 36 modification regulators of mRNA in the TCGA cohort, clustered by heatmap. Tumour stage, age, clinical T, N and M stage, gender, tumour grade and modification types were considered for clustering. **c** A risk prediction model containing 10 modification regulators was constructed via stepwise regression in R with both direction methods, and patients could be defined as the high-risk and low-risk groups based on their risk scores. The survival time of patients decreased as the risk score of the model increased. The heatmap was generated to visualize the expression of the 10 risk genes. **d** The mRNA levels of the high-risk group, low-risk group in HNSCC and healthy people are shown. **e** The overall survival of patients based on the risk prediction model. The high-risk group had a lower survival probability than the low-risk group (log-rank test, P < 0.0001)

To construct a model to predict the prognosis of patients, 36 modification regulators of mRNA were selected and analyzed (Additional file 1: Table S1). The workflow chart of the study is shown in Fig. 1a. The mRNA levels of these regulators in the TCGA cohort and their corresponding clinical information were visualized (Fig. 1b). Stepwise regression in R with both direction methods was applied, and a risk prediction model containing 10 modification regulators was constructed. The risk score of each patient was calculated. Patients were defined as the high-risk and low-risk groups based on their risk scores, and shorter survival time was observed with increasing risk score (Fig. 1c). In addition, a heatmap was generated to visualize the expression of the 10 risk genes (Fig. 1c). The mRNA levels of the 10 genes among the high-risk, low-risk and normal groups were also displayed (Fig. 1d). The OS analysis illustrated that the high-risk group had a lower survival probability than the low-risk group (Fig. 1e).

### NAT10 was a key risk gene and independent prognostic factor of HNSCC

**Fig. 2:**
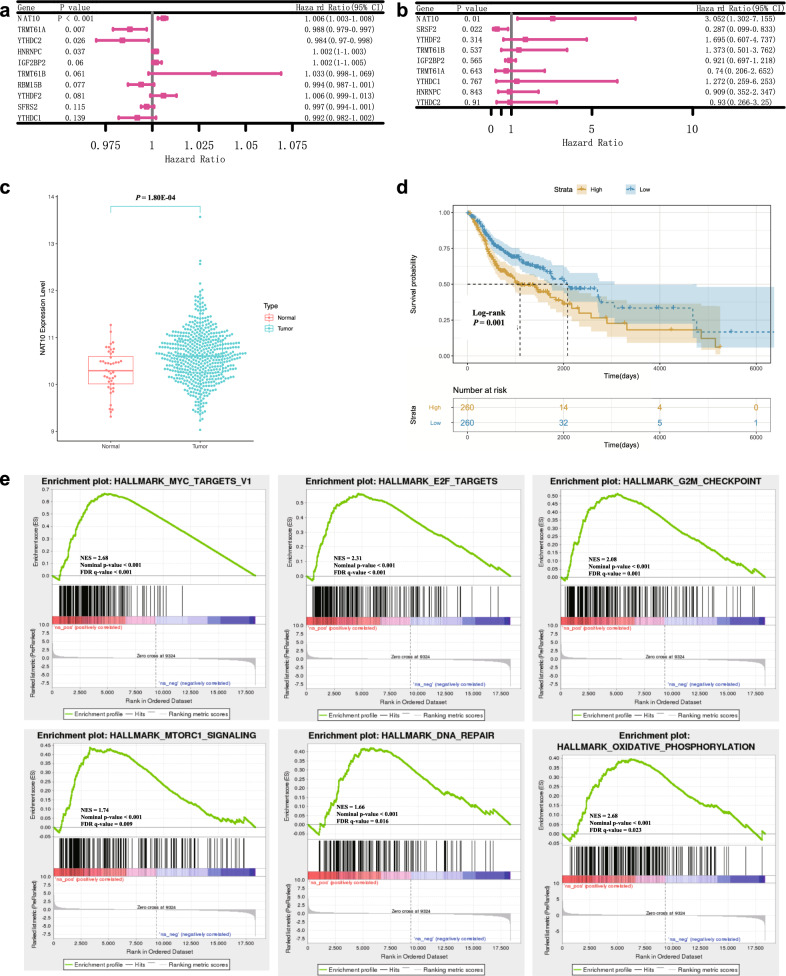
NAT10 is a key risk gene and independent prognostic factor of HNSCC in TCGA and GEO datasets. **a**, **b** NAT10, identified from TCGA and GEO data, was the most significant contributor to the hazard ratio of the model (RBM15B was not found in the GEO dataset). **c** In the TCGA cohort, the mRNA expression of NAT10 in tumour tissues was higher than that in normal tissues. **d** High expression of NAT10 was a poor prognostic factor for HNSCC (log-rank test, P = 0.001). **e** HNSCC samples were divided into NAT10-high and NAT10-low expression groups according to the median expression of NAT10. GSEA was performed, and the pathways associated with high NAT10 expression are shown

To further identify the most essential risk gene of the model, univariate Cox analysis was performed on TCGA and GEO datasets, and the hazard ratio of each gene was calculated (RBM15B was not found in the GEO dataset). The results indicated that NAT10 was the most significant gene with a higher hazard ratio among the 10 modification regulators (Fig. 2a, b). In addition, the expression of NAT10 was upregulated in HNSCC samples than in normal samples (Fig. 2c), and a lower OS probability was observed in the NAT10 high expression group of HNSCC patients (Fig. 2d). GSEA was performed to explore the enriched pathways associated with the high expression of NAT10. As shown in Fig. 2e, the gene sets were enriched in the MYC, E2F, G2M checkpoint, mTORC1, DNA repair and oxidative phosphorylation pathways. These pathways are associated with tumour progression, which indicates the essential role of NAT10 in HNSCC.

### High protein expression of NAT10 indicated worse OS in HNSCC patients

**Fig. 3:**
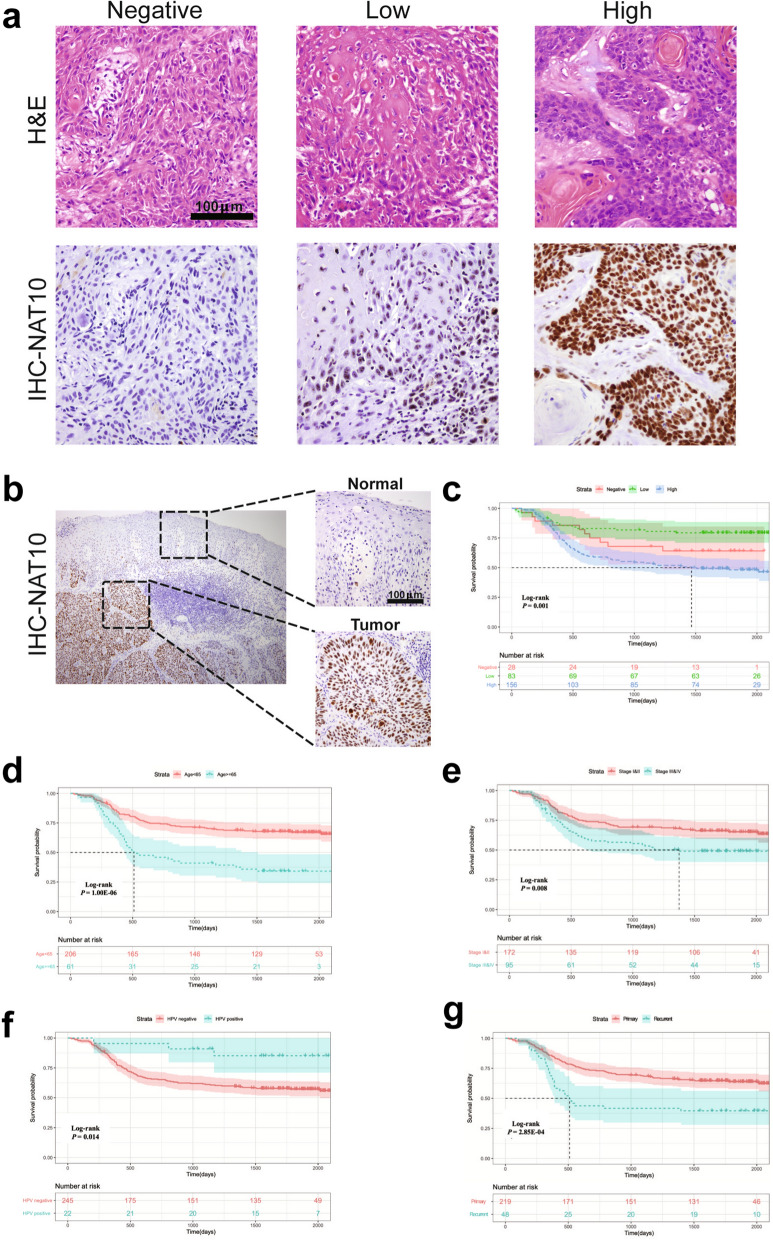
High expression of NAT10 indicated shorted OS in 267 patients according to IHC analysis. **a** Representative images of H&E and IHC staining of NAT10. **b** Representative images of NAT10 staining in tumour and normal epithelial cells. **c** The patients with high expression of NAT10 had a lower OS rate than those with low and no expression of NAT10 (log-rank test, P = 0.001; high expression of NAT10 was detected in 156 patients, low expression of NAT10 was detected in 83 patients, and no NAT10 expression was detected in 28 patients). **d** Older patients (greater than 65 years old) had a lower OS rate in our cohort (log-rank test, P < 0.001). **e** Patients with advanced stage (III and IV stages) had a lower OS rate (log-rank test, P = 0.008). **f** Patients infected with HPV had a higher OS rate (log-rank test, P < 0.014). **g** Tumour recurrence was an unfavorable factor for OS in HNSCC (log-rank test, P < 0.001)

To validate the prognostic role of NAT10 at the protein level, H&E and IHC staining of NAT10 from 267 HNSCC clinical FFPE samples were performed, and representative images of negative, low and high staining of NAT10 are shown (Fig. 3a). NAT10 was mainly located in the nucleus and more highly expressed in the tumour cells than that in normal epithelial cells (Fig. 3b). Based on the expression of NAT10, patients were divided into three groups (NAT10 negative, NAT10 low and NAT10 high group), and the clinical pathological parameters in these groups of patients were archived and compared with each other. We found that age (≥ 65), tumour site (oral cavity) and tumour type (recurrent) were strongly associated with NAT10 high group (Table 1). Univariate Cox regression survival analysis was performed to identify the risk factors for overall survival, and the results illustrated that NAT10 high group, age (≥ 65), tumour stage (III and IV), HPV-negative status, and recurrent tumour type were risk factors for OS (Additional file 2: Table S2). Low survival probability was found in patients with high expression of NAT10 (Fig. 3c), age ≥ 65 (Fig. 3d), stage III and IV disease (Fig. 3e), HPV-negative status (Fig. 3f) and recurrent tumours (Fig. 3g). Multivariate Cox regression survival analysis was further performed among the five mentioned risk factors above, and the results revealed that high expression of NAT10, age ≥ 65 and recurrent tumours were independent risk factors for HNSCC patients’ OS (Table 2).

**Table 1:** Clinical pathological parameters of the Chinese HNSCC cohort defined by the NAT10 expression signature

Clinical-pathological parameters	NAT10 negative	NAT10 low	NAT10 high	P value
Age				**0.001**
< 65	25	72	109	
≥ 65	3	11	47	
Gender				0.213
Male	24	48	95	
Female	4	35	61	
Site				**7.35E−09**
Oral cavity	10	64	141	
Oropharynx	18	19	15	
Smoking				0.058
No	15	55	114	
Yes	13	28	42	
Alcohol				0.476
No	19	67	124	
Yes	9	16	32	
Stage				0.149
I&II	20	57	95	
III&IV	8	26	61	
HPV				0.212
Negative	25	74	146	
Positive	3	9	10	
Grade				0.102
G1	15	40	59	
G2	10	36	84	
G3	3	7	13	
Type				**0.004**
Primary	26	74	119	
Recurrent	2	9	37

**Table 2:** Multivariate survival analyses of factors contributing to OS in Chinese HNSCC patients were performed using the Cox proportional hazards regression model

Clinical-pathological parameters	Patients (n = 267)	MST (days)	HR (95% CI)	P value
NAT10 IHC				**0.012**
Low	83	1872	Reference	
High	156	1378	1.564 (1.105–2.213)	
Negative	28	1419		
Age				**2.94E−05**
< 65	206	1738	Reference	
≥ 65	61	510	2.399 (1.591–3.616)	
Stage				0.068
I&II	172	1685	Reference	
III&IV	95	1177	1.435 (0.974–2.114)	
HPV				0.125
Positive	22	1664	Reference	
Negative	245	1569	2.475 (0.776–7.874)	
Type				**0.005**
Primary	219	1625	Reference	
Recurrent	48	511	1.898 (1.211–2.974)	

### Downregulation of NAT10 inhibited the proliferation ability of three HNSCC cell line cells

**Fig. 4:**
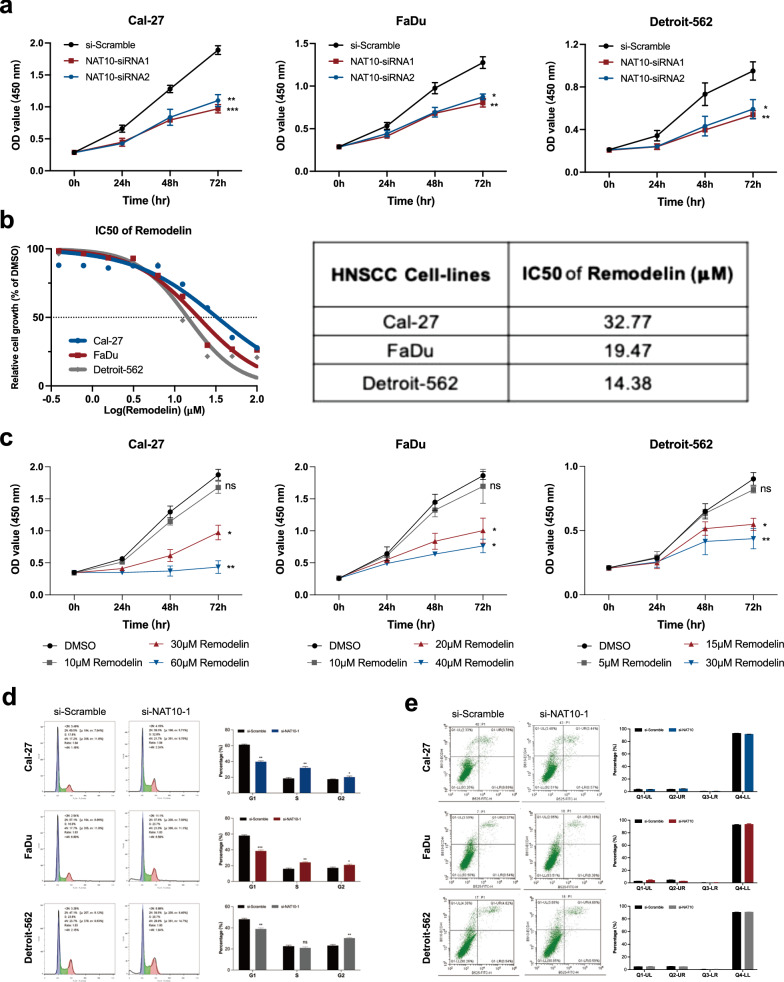
Downregulation of NAT10 inhibited the proliferation of Ca-l27, FaDu and Detroit-562 cell lines and arrested them in S/G2 phase in vitro. **a** The proliferation rates of Cal-27, FaDu and Detroit-562 cell lines were decreased by the two siRNAs of NAT10. **b** The IC50 values of Remodelin (an inhibitor of NAT10) were calculated in three cell lines by CCK-8 assay. **c** Remodelin decreased the proliferation rates of Cal-27, FaDu and Detroit-562 cell lines. **d** The cell cycle of the three cell lines was arrested in S and G2 phases by NAT10 siRNA, and data were analyzed by FlowJo software. **e** Three cell lines were transfected with NAT10 siRNA in vitro, and the results indicated no difference in cell apoptosis. All statistical analyses were performed with SPSS 23.0. T tests were performed to compared differences between two groups, and one-way ANOVA was performed to compare differences between three or more groups. ns: no significance, *P < 0.05, **P < 0.01, ***P < 0.001, ****P < 0.0001

Three siRNAs of NAT10 and si-Scramble were constructed, and their knockdown efficacy in three HNSCC cell lines (Cal-27, FaDu and Detroit-562) was verified by qRT-PCR and western blotting (Additional file 3: Fig. S1). To reveal the role of NAT10 in cell proliferation ability, the three cell lines were treated with two siRNAs of NAT10, and si-Scramble was administered as a control. Reduced proliferation ability was observed after treating the cell lines with siRNAs (Fig. 4a). In addition, Remodelin as a chemical inhibitor of NAT10 was applied at different concentrations and the CCK-8 assay indicated that Remodelin inhibited the proliferation of HNSCC cells. The half-maximal inhibitory concentrations (IC50s) of Remodelin ranged from 14.38 to 32.77 µM (Fig. 4b). Remodelin significantly inhibited the proliferation ability, and proliferation rates were decreased as the concentration were increased (Fig. 4c). Cell cycles were arrested in the S and G2 stages by NAT10 siRNA (Fig. 4d). However, there was no difference in cell apoptosis between the si-Scramble and si-NAT10 groups (Fig. 4e).

### NAT10 inhibition decreased the migration and invasion abilities of HNSCC cell lines

**Fig. 5:**
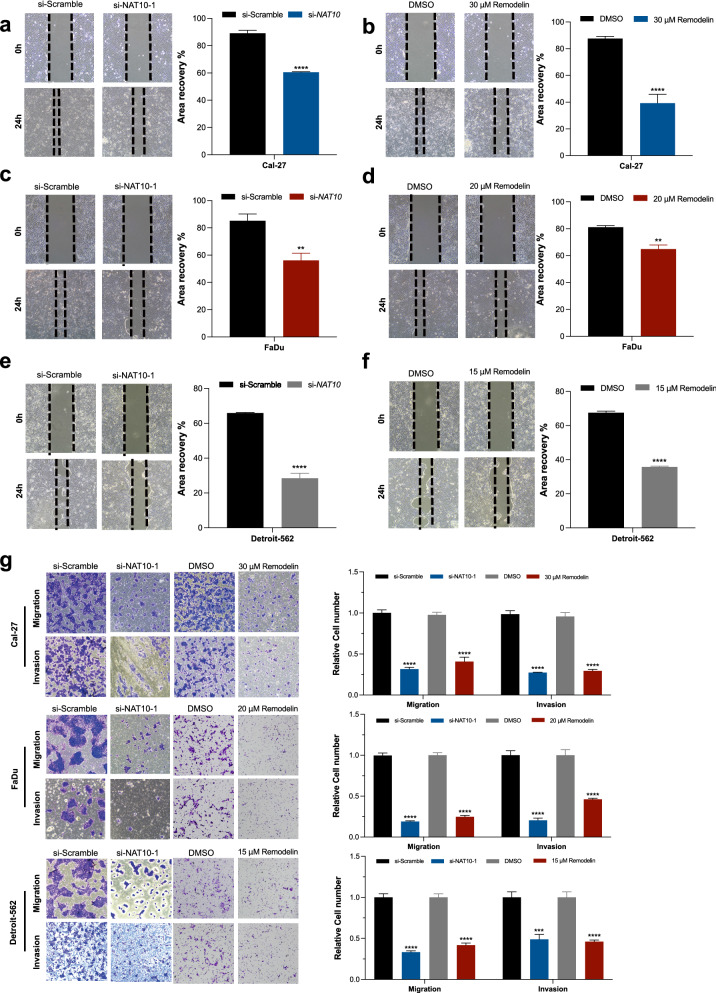
Both genetic and pharmacologic inhibition of NAT10 suppressed the migration and invasion in human HNSCC cells. **a**–**f** A cell scratch assay was performed, and three cell lines were treated with either Remodelin or siRNA. Representative figures are shown. ImageJ was used to calculate the area without cells, and the results indicated that the cell migration abilities were decreased after NAT10 was inhibited. **g** For the migration assay, cell lines were treated with siRNA targeting NAT10 or Remodelin and approximately 10^5^ cells were seeded in the apical chamber of the Transwell. Crystal violet staining was performed 48 h later. The migration ability of the three cell lines was decreased as shown. For the invasion assay, the apical chamber of the Transwell was precoated with Matrigel. Cell lines were treated with siRNA targeting NAT10 or Remodelin, and approximately 10^5^ cells were seeded in the apical chamber of the Transwell. Crystal violet staining indicated decreased invasion ability of the three cell lines after NAT10 was inhibited. A t test was used to compare two groups for statistical analysis, *P < 0.05, **P < 0.01, ***P < 0.001, ****P < 0.0001

Tumour cell migration and invasion are essential for cancer progression. A cell scratch assay was used to evaluate the horizontal migration ability. Our results demonstrated that the migration ability was decreased by treatment of Remodelin and NAT10 siRNA (Fig. 5a–f). Transwell chambers were used in the cell migration and invasion assays. For the cell invasion assay, the apical chamber was precoated with Matrigel. Cell lines were treated with Remodelin or NAT10 siRNA. Approximately 10^5^ cells were seeded in the apical chamber of the transwell. Crystal violet staining revealed that the migration ability and invasion ability of HNSCC cells were decreased after NAT10 was inhibited (Fig. 5g).

### Remodelin inhibited the growth of HNSCC in PDX model

**Fig. 6:**
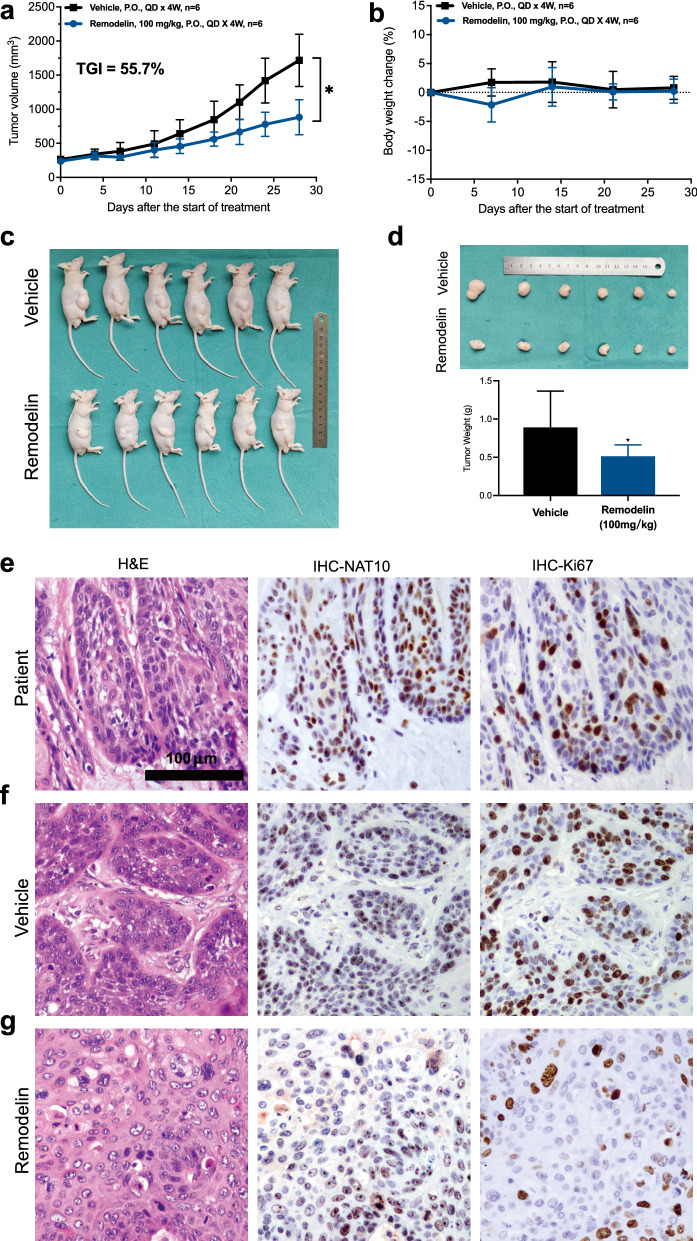
Remodelin inhibited the growth of HNSCC in the PDX model. **a**, **b** Nude mice were used for PDX model construction, Remodelin (100 mg/kg) was given daily by intragastric administration in the treatment group, and vehicle was given as a control. Tumour volume was recorded every 3–5 days, and the mice were sacrificed after intragastric administration for 4 weeks. The results indicated that HNSCC could be inhibited by Remodelin and that the body weights of mice were not affected (T test, *P < 0.05, **P < 0.01, ***P < 0.001, ****P < 0.0001). **c**, **d** Images of mice after Remodelin administration for 4 weeks. Tumour weight was decreased in the treatment group (T test, *P < 0.05). **e** HE and IHC staining of tumours from patients. NAT10 and Ki-67 were highly expressed. **f** HE and IHC staining of tumours from the PDX model without Remodelin administration. NAT10 and Ki-67 were highly expressed. **g** HE and IHC staining of tumours from the PDX model with Remodelin administration, lower expression of Ki-67 was observed

To evaluate the therapeutic value of Remodelin in HNSCC in vivo, PDX models were constructed. Remodelin was given daily by intragastric administration for 4 weeks. Tumour volume was recorded and TGI was calculated. As a result, Remodelin significantly inhibited the growth of HNSCC PDXs in vivo. The TGI was 55.7%, and the mice’s body weights were not affected (Fig. 6a, b). In addition, the tumour weight was significantly decreased in the treatment group compared with the vehicle group (Fig. 6c, d). IHC staining were performed to detect the expression of NAT10 and Ki-67 in tumours from patients (Fig. 6e), the PDX model without Remodelin administration (Fig. 6f), and the PDX model with Remodelin administration (Fig. 6g). As shown in the pictures, lower expression of Ki-67 was observed in the Remodelin treatment group, which indicated that Remodelin inhibited tumour proliferation ability in vivo.

### Remodelin regulated the expression of MYC and LDHA

**Fig. 7:**
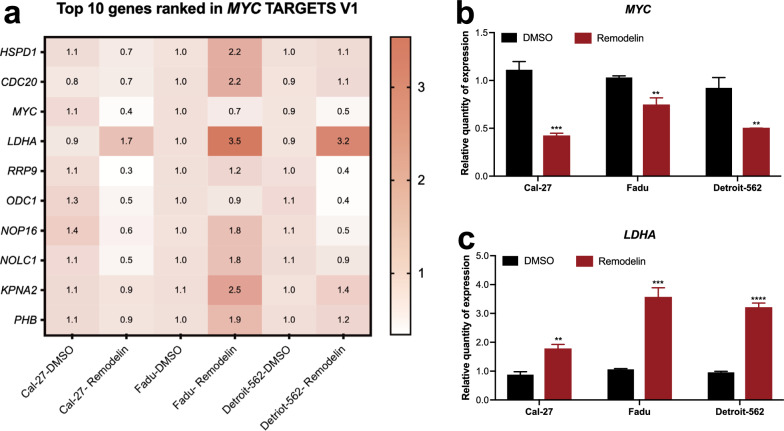
Remodelin regulated the expression of MYC and LDHA. **a** Over view of the relative quantity expression of the top 10 ranked genes in MYC targets after treated with Remodelin. **b** MYC was significantly downregulated in all three cell lines by Remodelin. **c** LDHA was upregulated after NAT10 was inhibited by Remodelin

MYC targets is the most enriched pathway in GSEA results, qRT-PCR was performed to evaluate the expression of the top 10 enriched genes of MYC targets in three cell lines after treated with Remodelin for 48 h (Fig. 7a). Among the top 10 genes, MYC was significantly downregulated by Remodelin, but the expression of LDHA was upregulated in all three cell lines (Fig. 7b, c). The results indicated MYC and LDHA regulation was associated with the inhibition of NAT10.

## Discussion

It is clear that the mRNA modifications and related regulators play critical roles in tumorigenesis and progression of various cancers [13]. In this study, we firstly applied bioinformatic methods to construct a risk prediction model containing 10 mRNA modification regulators related to six reported distinct forms of mRNA modifications. Moreover, survival analysis of this prediction model showed that prognosis of the high-risk group had a lower OS survival probability than the low-risk group. Based on univariate Cox analysis performed on TCGA and GEO datasets, NAT10 was identified as an independent essential gene in this risk model. In addition, we further identified NAT10 as an independent prognostic factor associated with the OS of HNSCC patients in our clinical cohort. Furthermore, the cell proliferation, migration and invasion abilities of three HNSCC cell lines were inhibited by genetic depletion of NAT10 using siRNA or chemical inhibitor, Remodelin. Knockdown of NAT10 using siRNA increased cell cycle arrest in S/G2-phase. Finally, the therapeutic value of Remodelin was evaluated in a HNSCC PDX model and found to inhibit the growth of tumours.

It is of great significance to identify risk models and biomarkers for predicting the prognosis of patients and the selection of appropriate clinical strategies [27]. In our study, patients with HNSCC could be stratified into a high-risk cohort with worse OS and a low-risk cohort with better OS based on our risk prediction model in TCGA dataset. Univariate Cox analysis further revealed that NAT10 was the most significant gene with the highest hazard ratio among these model signatures in TCGA and GEO datasets. NAT10 (*N*-acetyltransferase 10) is a histone acetyltransferase associated with many biological processes mainly distributed in the nucleolus [28–30]. With acetyltransferase and RNA binding activities, NAT10 is the only human enzyme to catalyze ac4C production in eukaryotic RNA [31]. In-depth studies demonstrated that ac4C as an mRNA modification plays a key role in controlling telomerase activity, the synthesis of rRNA, the repair of DNA damage and the stabilization of mRNA. It is also related to the development, progression, and prognosis of various types of human diseases, especially cancer. [16, 31, 32]. As the only ac4C modification producer, NAT10 is significantly associated with the prognosis of several malignant tumours and potently promotes the tumour progression [30, 33–39]. High expression of NAT10 in hepatocellular carcinoma [30, 37, 39] and colorectal cancer [38] is associated with a worse prognosis in patients. However, few studies have assessed the role of NAT10 in HNSCC. Based on public data from the TCGA and 267 HNSCC samples in our cohort, we identified NAT10 as a potential prognostic biomarker in HNSCC patients. In this study, NAT10 was more highly expressed in HNSCC tissues than in normal tissues, and that upregulated expression of NAT10 indicated worse OS of HNSCC. In hepatocellular carcinoma, the tumorigenic mechanism involves the redistribution of NAT10 from nucleoli to karyotheca, the cytoplasm and the cell membrane, and NAT10 can upregulate mutant p53 (a tumour suppressor gene) levels [30, 37, 39]. In addition, laryngeal cancer cell migration was reported to be promoted by the MYCT1/NAT10 axis [35]. Likewise, MYC targets had the highest normalized enrichment score (NES) among all the gene sets in our GSEA results, and qRT-PCR results of the top 10 ranked genes of MYC targets indicated Remodelin downregulated the expression of MYC and upregulated the expression of LDHA in HNSCC cell lines.

Increasing evidence indicated that NAT10 might be a potential therapeutic target for cancer treatment [40–43]. Therefore, we analyzed the therapeutic potential of NAT10 in HNSCC and observed that the proliferation, migration and invasion abilities of HNSCC cell lines were decreased after NAT10 was inhibited by siRNA and Remodelin in this study. Besides, our result showed that the cell cycle of the three HNSCC cell lines was arrested in the G2/S stage after downregulation of NAT10 by siRNA. Interestingly, our GSEA results also revealed gene sets in the NAT10 high expression group involved in the G2/M checkpoint and associated with DNA repair. NAT10-mediated acetylation of MORC2 could force the cell to pass through the G2 checkpoint and confer resistance to DNA-damaging treatments in a breast cancer study [36]. Furthermore, our in vivo study yielded preclinical data relevant to the treatment of HNSCC in the clinic. Specifically, Remodelin inhibited the growth of a HNSCC-PDX model with high expression of NAT10. Remodelin is a small-molecule compound explored as potent inhibitor of NAT10 and firstly reported that targeting NAT10 might be an alternative strategy for the treatment of laminopathies and aging [25]. More recently, Remodelin showed a reverse effect on cell proliferation, cell invasion, and migration of several tumours [37, 43]. Although the research of Remodelin is still at a very early stage, our results indicate that NAT10 may be a promising drug target. Moreover, inhibition of NAT10 might be a new therapeutic strategy for HNSCC. We anticipate that Remodelin could be examined as a drug for HNSCC in clinical trials in the very near future.

Tumour cell proliferation, migration, invasion, and G2/S stage promotion were proven to be regulated by NAT10, thus aggravating the progression of HNSCC in the present study. However, one limitation of our study is that the other GSEA results need to be verified in further studies. For example, the specific role of NAT10 in E2F target, and mTORC1 signalling in HNSCC needs in-depth research. In addition, oxidative phosphorylation is essential for tumour growth [44] and it is necessary to explore whether NAT10 contributes to the metabolism of tumours. Moreover, targeting NAT10 alone or in combination with other drugs may be a new strategy for tumour treatment in further investigations.

Above all, this study reported the construction of a risk prediction model and the identification of NAT10 as a critical tumour growth-favoring factor in HNSCC. High expression of NAT10 was associated with an unfavorable prognosis in HNSCC patients in the TCGA cohort and was verified in our clinical cohort, which included 267 patients. NAT10 promoted tumour growth by regulating the migration, invasion and cell cycle of tumour cells. Remodelin, an inhibitor of NAT10, significantly suppressed the growth of HNSCC in a PDX model, indicating that Remodelin may be a new candidate drug for HNSCC treatment.

## Conclusions

In conclusion, NAT10 was a key gene in HNSCC development and progression. As verified by IHC results of 267 HNSCC samples, NAT10 is associated with the poor prognosis in HNSCC patients. In addition, in vitro and in vivo studies proved that NAT10 could potentially be the therapeutic target in HNSCC. The migration, invasion and cell cycle promotion of the tumour cells may be the mechanisms by which NAT10 promotes tumour progression.

## Supplementary Information

**Additional file 1: Table S1.** The 36 modification regulators of mRNA.

**Additional file 2: Table S2.** Univariate survival analyses of factors contributing to OS in Chinese HNSCC patients.

**Additional file 3: Figure S1.** The expression of NAT10 was inhibited at the RNA and protein levels by siRNA. (a) qRT-PCR was used to verify the expression of NAT10. After transfection with three siRNAs, the expression of NAT10 was downregulated in the three cell lines. (b) Western blotting was applied to verify the expression of NAT10 at the protein level. NAT10 was strongly expressed in cells transfected with si-Scramble, while NAT10 was expressed at low levels in cells transfected with si- NAT10-1 and si- NAT10-2.

## Data Availability

The datasets generated and/or analyzed during the current study are available in GEO (http://www.ncbi.nlm.nih.gov/geo) and TCGA through firebrowse (http://firebrowse.org/?cohort=HNSC&download_dialog=true#) website.

## Abbreviations

ac4C: N4-acetylcytidine
ATCC: American Type Culture Collection
CCK8: Cell Counting Kit-8
DMEM: Dulbecco’s Modified Eagle Medium
DMSO: Dimethyl sulfoxide
FAM: 5-Carboxyfluorescein
FFPE: Formalin fixed paraffin-embedded
GEO: Gene Expression Omnibus
GSEA: Gene set enrichment analysis
HE: Hematoxylin and eosin
HNSCC: Head and neck squamous cell carcinoma
HPV: Human papilloma virus
IC50: Half maximal inhibitory concentration
IHC: Immunohistochemistry
m1A: N1-methyladenosine
m3C: 3-Methylcytosine
m5C: 5-Methylcytosine
m6A: N6-methyladenosine
MORC2: Microrchidia family CW-type zinc finger 2
MRs: Modification regulators
MYCT1: MYC target 1
NAT10: *N*-acetyltransferase 10
NES: Normalized enrichment score
OD: Optical density
OS: Overall survival
PVDF: Polyvinylidene fluoride
PDX: Patient-derived xenografts
PI: Propidium iodide
qRT-PCR: Quantitative real-time polymerase chain reaction
SD: Standard deviation
TCGA: The Cancer Genome Atlas
TGI: Tumour growth inhibition rate
TV: Tumour volume
