# Supplementary material for: NAT10 as a potential prognostic biomarker and therapeutic target for HNSCC
Source: Cancer Cell Int. 2021 Aug 6;21:413. doi: 10.1186/s12935-021-02124-2 (PMC8344148; doi:10.1186/s12935-021-02124-2)
Supplement: Supplementary file 1 — Additional file 1: Table S1. The 36 modification regulators of mRNA. [file 12935_2021_2124_MOESM1_ESM.docx]

**Additional file 1: Table S1. The 36 modification regulators of mRNA.**

| **Regulator** | **Genes** | **Aliases** | **Type** |
| --- | --- | --- | --- |
| m6A Writers | RBM15 | RBM15 | m6A |
| m6A Writers | RBM15B | RBM15B | m6A |
| m6A Writers | METTL3 | METTL3 | m6A |
| m6A Writers | METTL14 | METTL14 | m6A |
| m6A Writers | WTAP | WTAP | m6A |
| m6A Writers | VIRMA | KIAA1429 | m6A |
| m6A Readers | SRSF2 | SFRS2 | m6A |
| m6A Readers | RBMX | RBMX | m6A |
| m6A Readers | ZC3H13 | ZC3H13 | m6A |
| m6A Readers | YTHDC1 | YTHDC1 | m6A |
| m6A Readers | YTHDC2 | YTHDC2 | m6A |
| m6A Readers | YTHDF1 | YTHDF1 | m6A |
| m6A Readers | YTHDF2 | YTHDF2 | m6A |
| m6A Readers | YTHDF3 | YTHDF3 | m6A |
| m6A Readers | IGF2BP1 | IGF2BP1 | m6A |
| m6A Readers | IGF2BP2 | IGF2BP2 | m6A |
| m6A Readers | IGF2BP3 | IGF2BP3 | m6A |
| m6A Readers | HNRNPA2B1 | HNRNPA2B1 | m6A |
| m6A Readers | HNRNPC | HNRNPC | m6A |
| m6A Erasers | FTO | FTO | m6A |
| m6A Erasers | ALKBH5 | ALKBH5 | m6A |
| m5C Writers | NSUN2 | NSUN2 | m5C |
| m5C Writers | DNMT2 | TRDMT1 | m5C |
| m5C Writers | TET1 | TET1 | m5C |
| m5C Writers | TET2 | TET2 | m5C |
| m5C Writers | TET3 | TET3 | m5C |
| m5C Readers | ALYREF | THOC4 | m5C |
| m1A Writers | TRMT6 | TRMT6 | m1A |
| m1A Writers | TRMT61A | TRMT61A | m1A |
| m1A Writers | TRMT61B | TRMT61B | m1A |
| m1A Writers | TRMT10C | RG9MTD1 | m1A |
| m1A Erasers | ALKBH3 | ALKBH3 | m1A |
| 𝚿 Writer | PUS1 | PUS1 | 𝚿 |
| 𝚿 Writer | PUS7 | PUS7 | 𝚿 |
| m3C | METTL8 | METTL8 | m3C |
| ac4C | NAT10 | NAT10 | ac4C |
