# Supplementary material for: NAT10 as a potential prognostic biomarker and therapeutic target for HNSCC
Source: Cancer Cell Int. 2021 Aug 6;21:413. doi: 10.1186/s12935-021-02124-2 (PMC8344148; doi:10.1186/s12935-021-02124-2)
Supplement: Supplementary file 2 — Additional file 2: Table S2. Univariate survival analyses of factors contributing to OS in Chinese HNSCC patients. [file 12935_2021_2124_MOESM2_ESM.docx]

**Additional file 2: Table S2.** Univariate survival analyses of factors contributing to OS in Chinese HNSCC patients.

| **Clinical-pathological Parameters** | **Patients （n=267）** | **MST**  **(days)** | **HR**  **(95% CI)** | **P value** |
| --- | --- | --- | --- | --- |
| **Tumor NAT10 IHC** |  |  |  | 0.001 |
| **Low** | 83 | 1872 | Reference |  |
| **High** | 156 | 1378 | 3.007 (1.781-5.077) |  |
| **Negative** | 28 | 1419 |  |  |
| **Age** |  |  |  | **1.00E-06** |
| **<65** | 206 | 1738 | Reference |  |
| **>=65** | 61 | 510 | 2.634 (1.778-3.904) |  |
| **Gender** |  |  |  | 0.894 |
| **Male** | 167 | 1570 | Reference |  |
| **Female** | 100 | 1582 | 1.027 (0.698-1.512) |  |
| **Site** |  |  |  | 0.389 |
| **Oral cavity** | 215 | 1625 | Reference |  |
| **Oropharynx** | 52 | 1372 | 0.800 (0.482-1.328) |  |
| **Smoking** |  |  |  | 0.977 |
| **No** | 184 | 1586 | Reference |  |
| **Yes** | 83 | 1562 | 1.006 (0.670-1.509) |  |
| **Alcohol** |  |  |  | 0.802 |
| **No** | 210 | 1596 | Reference |  |
| **Yes** | 57 | 1557 | 1.059 (0.673-1.668) |  |
| **Stage** |  |  |  | **0.008** |
| **I&II** | 172 | 1685 | Reference |  |
| **III&IV** | 95 | 1177 | 1.668 (1.141-2.439) |  |
| **HPV** |  |  |  | **0.014** |
| **Positive** | 22 | 1664 | Reference |  |
| **Negative** | 245 | 1569 | 3.797(1.205-1.965) |  |
| **Grade** |  |  |  | 0.051 |
| **G1** | 114 | 1608 | Reference |  |
| **G2** | 130 | 1596 | 1.237 (0.823-1.859) |  |
| **G3** | 23 | 1077 | 1.352 (0.989-1.849) |  |
| **Type** |  |  |  | **2.85E-04** |
| **Primary** | 219 | 1625 | Reference |  |
| **Recurrent** | 48 | 511 | 2.168 (1.414-3.323) |  |
