# Supplementary figures and images for: NAT10 as a potential prognostic biomarker and therapeutic target for HNSCC
Source: Cancer Cell Int. 2021 Aug 6;21:413. doi: 10.1186/s12935-021-02124-2 (PMC8344148; doi:10.1186/s12935-021-02124-2)

**a**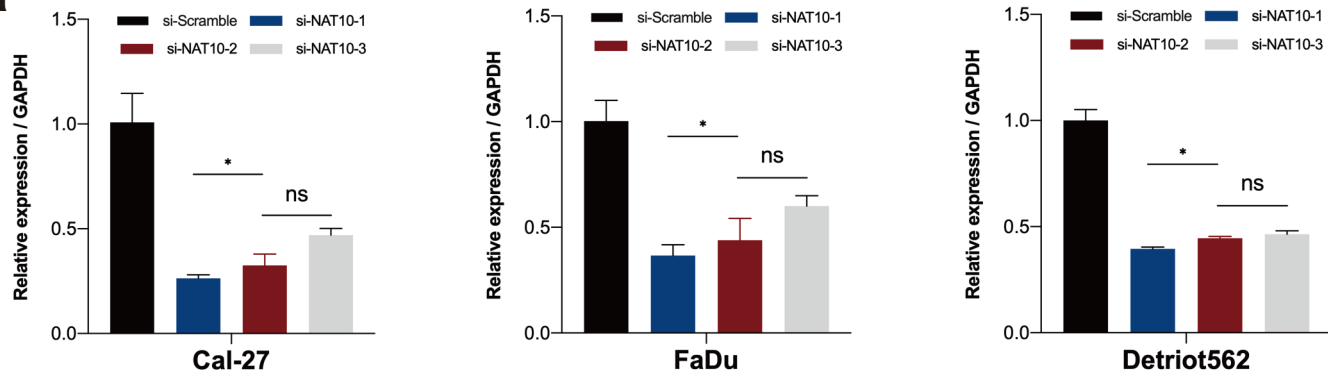**b**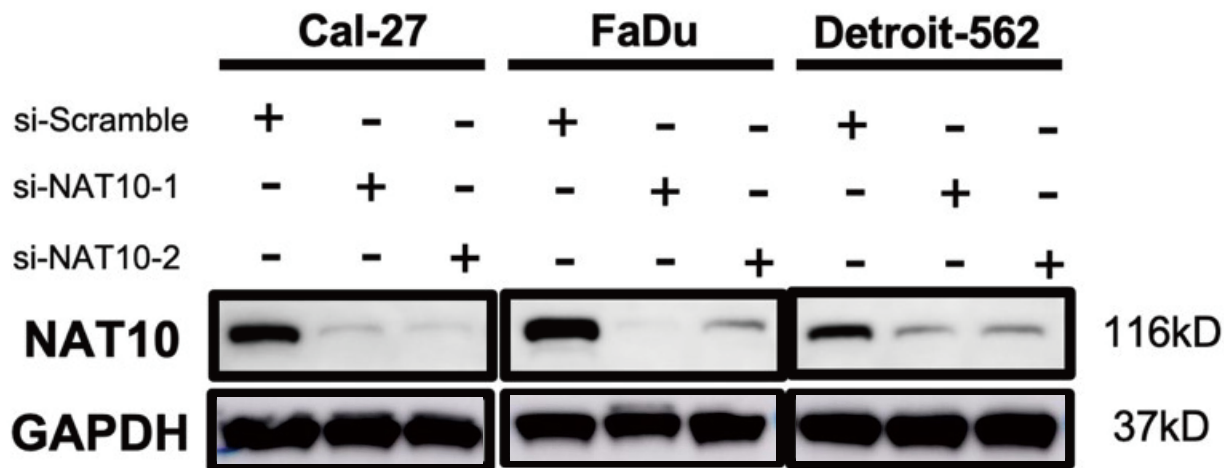

Supplement: Supplementary file 3 — Additional file 3: Figure S1. The expression of NAT10 was inhibited at the RNA and protein levels by siRNA. (a) qRT-PCR was used to verify the expression of NAT10. After transfection with three siRNAs, the expression of NAT10 was downregulated in the three cell lines. (b) Western blotting was applied to verify the expression of NAT10 at the protein level. NAT10 was strongly expressed in cells transfected with si-Scramble, while NAT10 was expressed at low levels in cells transfected with si- NAT10-1 and si- NAT10-2. [file 12935_2021_2124_MOESM3_ESM.pdf]
